# Ultrasonography of Quadriceps Femoris Muscle and Subcutaneous Fat Tissue and Body Composition by BIVA in Chronic Dialysis Patients

**DOI:** 10.3390/nu12051388

**Published:** 2020-05-12

**Authors:** Yuri Battaglia, Ines Ullo, Sara Massarenti, Pasquale Esposito, Michele Prencipe, Giovanni Ciancio, Michele Provenzano, Fulvio Fiorini, Michele Andreucci, Alda Storari, Alice Sabatino, Enrico Fiaccadori, Antonio Granata, On behalf of the Ultrasound Study Group of the Italian Society of Nephrology

**Affiliations:** 1 Division of Nephrology and Dialysis, St. Anna University Hospital, 44121 Ferrara, Italy; a.storari@ospfe.it; 2 Division of Nephrology, ASST Sette Laghi, 21100 Varese, Italy; inesullo@gmail.com; 3 Department of Biomedical and Specialty Surgical Sciences, University of Ferrara, 44121 Ferrara, Italy; massarenti.sara85@gmail.com; 4 Division of Nephrology, Dialysis and Transplantation, University of Genoa and IRCCS Ospedale Policlinico San Martino, 16132 Genoa, Italy; pasqualeesposito@hotmail.com; 5 Division of Nephrology, Casa Sollievo della Sofferenza, San Giovanni Rotondo, 71100 Foggia, Italy; mikprenc@libero.it; 6 Division of Rheumatology, University of Ferrara, 44121 Ferrara, Italy; g.ciancio@ospfe.it; 7 Division of Nephrology and Dialysis, Department of Health Sciences, Magna Graecia University, 88100 Catanzaro, Italy; michiprov@hotmail.it (M.P.); andreucci@unicz.it (M.A.); 8 Division of Nephrology and Dialysis, “Santa Maria della Misericordia” Hospital, 45100 Rovigo, Italy; fulvio.fiorini@aulss5.veneto.it; 9 Nephrology Unit, Department of Medicine and Surgery, Parma University Hospital, 43121 Parma, Italy; alice.sabatino86@gmail.com (A.S.); enrico.fiaccadori@unipr.it (E.F.); 10 Division of Nephrology, San Giovanni di Dio Hospital, 92100 Agrigento, Italy; antonio.granata@tin.it

**Keywords:** BIVA, fat tissue, muscle mass, PEW, ultrasonography

## Abstract

Protein Energy Wasting (PEW) in hemodialysis (HD) patients is a multifactorial condition due to specific pathology-related pathogenetic mechanisms, leading to loss of skeletal muscle mass in HD patients. Computed Tomography and Magnetic Resonance Imaging still represent the gold standard techniques for body composition assessment. However, their widespread application in clinical practice is difficult and body composition evaluation in HD patients is mainly based on conventional anthropometric nutritional indexes and bioelectrical impedance vector analysis (BIVA). Little data is currently available on ultrasound (US)-based measurements of muscle mass and fat tissue in this clinical setting. The purpose of our study is to ascertain: (1) if there are differences between quadriceps rectus femoris muscle (QRFM) thickness and abdominal/thigh subcutaneous fat tissue (SFT) measured by US between HD patients and healthy subjects; (2) if there is any correlation between QRFM and abdominal/thigh SFT thickness by US, and BIVA/conventional nutritional indexes in HD patients. We enrolled 65 consecutive HD patients and 33 healthy subjects. Demographic and laboratory were collected. The malnutrition inflammation score (MIS) was calculated. Using B-mode US system, the QRFM and SFT thicknesses were measured at the level of three landmarks in both thighs (superior anterior iliac spine, upper pole of the patella, the midpoint of the tract included between the previous points). SFT was also measured at the level of the periumbilical point. The mono frequency (50 KHz) BIVA was conducted using bioelectrical measurements (Rz, resistance; Xc, reactance; adjusted for height, Rz/H and Xc/H; PA, phase angle). 58.5% were men and the mean age was 69 (SD 13.7) years. QRFM and thigh SFT thicknesses were reduced in HD patients as compared to healthy subjects (*p* < 0.01). Similarly, also BIVA parameters, expression of lean body mass, were lower (*p* < 0.001), except for Rz and Rz/H in HD patients. The average QRFM thickness of both thighs at top, mid, lower landmarks were positively correlated with PA and body cell mass (BCM) by BIVA, while negatively correlated with Rz/H (*p* < 0.05). Abdominal SFT was positively correlated with PA, BCM and basal metabolic rate (BMR) (*p* < 0.05). Our study shows that ultrasound QRFM and thigh SFT thicknesses were reduced in HD patients and that muscle ultrasound measurements were significantly correlated with BIVA parameters.

## 1. Introduction

Protein energy wasting (PEW) is a condition peculiar to the most advanced stages of chronic kidney disease (CKD), and especially hemodialysis (HD) patients [1,2], and is associated with increased morbidity and mortality [3,4,5]. PEW is characterized by inadequate intake of nutrients, loss of energy reserves, derangements in body composition, and increased muscle protein catabolism, leading to lean body mass loss [6,7]. According to the International Society of Renal Nutrition and Metabolism (ISRNM) [8], reduced total body fat and muscle mass are relevant indicators for the diagnosis of PEW. 

Currently, Computed Tomography (CT) and Magnetic Resonance Imaging (MRI) are the gold standard for the assessment of body composition [9], but they are expensive, not practical nor always available in clinical practice, technically complex and, at least in the case of CT, it exposes patients to excess radiation [10]. 

Other methods such as dual-energy X-ray absorptiometry (DEXA) and bio-impedance analysis (BIA) have been used to assess body composition [11]. However, these methods can be inaccurate since they can be confounded by fluid status [12]. Other nutritional parameters, such as anthropometry and biochemical analysis are considered surrogates of muscle and fat mass; however, they do not provide enough information to allow for an accurate assessment of body composition status, because of the high variability of fluid status in HD patients and the presence of inflammation [13].

Considering all the limitations and pitfalls of the available tools for the assessment of body composition, the application of ultrasound technique to skeletal muscle could represent an interesting alternative tool and gained considerable interest in the last few years [14]. In critically ill patients, quadriceps femoris muscle mass is a very important parameter of muscularity, and its quantification is an indicator of lean body mass status and is not influenced by rapid fluid shifts [15,16]. In HD patients, quadriceps US was able to identify patients with worse nutritional status [17]. Moreover, the reliability and validity of the method as an alternative tool for the assessment of muscle mass was recently demonstrated also in patients with acute kidney injury [15,18]. Similarly, abdominal ultrasound for the assessment of visceral fat and quadriceps ultrasound for the assessment of subcutaneous fat also resulted reliable compared to DEXA, the reference technique, in medical settings [19,20,21].

The present study was aimed at investigating: (a) whether any difference exists in quadriceps rectus femoris muscle (QRFM) thickness and the abdominal/thigh subcutaneous fat tissue (SFT) thickness measured by US between HD patients and healthy subjects; (b) whether any correlation exist between QRFM, abdominal and thigh SFT, on one hand and bioelectrical impedance vector analysis (BIVA) parameters and conventional anthropometric and biochemical indexes of nutritional status in HD patients on the other hand.

## 2. Materials and Methods

A cross-sectional, observational, single center study was performed at the Hemodialysis Center of the Ferrara University Hospital. The study was approved by the Local Institutional Review Board (Ref No. 170192, 13 April 2017). The procedures were in agreement with the Declaration of Helsinki and written informed consent was obtained from all the participants.

### 2.1. Patients

Ninety-eight consecutive patients receiving chronic HD treatment were evaluated from June 2017 to December 2018. Exclusion criteria were limb amputation; prolonged hospitalization within the previous 30 days; bedridden or immobilization syndrome; dialysis vintage less than six months; presence of cardiac pacemaker, implantable cardioverter-defibrillator, or metallic non-removable pieces. 

Thirty-three healthy subjects from the hospital staff were enrolled. Inclusion criteria were age >18 years and absence of chronic or acute diseases.

In HD patients, demographic, clinical and anthropometric data were collected, and routine biochemistry was measured at the time of US and BIVA measurement. Daugirdas’ formula was used for standard Kt/V urea calculation, a DOQI-approved method [22]. Body mass index (BMI) was calculated as weight (kg)/height^2^ (m^2^).

### 2.2. Ultrasound Technique

QRFM thickness was measured using B-mode US system (Philips Envisor C HD) and 7.5 MHz linear array transducer, by an expert nephrologist at the patients’ bedside. The transducer was placed perpendicular to the long axis of the thigh, with minimal pressure to avoid compression of the muscle. Patients were laid down in a supine position with both knees extended but relaxed and toes pointing upwards, with legs forming a 45 °C angle.

QRFM thickness was measured in both thighs as the distance between the inferior surface of the fascia and the superior surface of the vastus femoris muscle. Measurements were performed at the level of three landmarks: the superior anterior iliac spine (top); the upper pole of the patella (lower); the midpoint of the tract included between the previous points (mid). 

The thickness of the peripheral SFT was measured at the same three landmarks of the both thighs while abdominal SFT was measured at the level of the xiphopubic line above the umbilicus, calculating the distance between the inferior surface of the derma and the superior surface of the most superficial muscular fascia. The measurement was repeated two times and the average value was used in the analyses. Assessor performed a total of 26 measurements (12 for QRFM and 14 for SFT) in each subject. 

### 2.3. Body Composition Evaluation

The monofrequency (50 KHz) BIVA (AKERN EFG Plus^®^, Pontassieve, FI, USA, with hydrasite technology) was used to obtain Rz, resistance; Xc, reactance; adjusted for height, Rz/H and Xc/H; and PA, phase angle) [23]. Body cell mass (BCM), extra-cellular mass (ECW), basal metabolic rate (BMR) were estimated from BIVA parameters [24]. 

Two electrodes, placed at a distance >5 cm, were attached to the same side arm and leg and to the opposite side of the arteriovenous fistula in supine patients. Measurements were performed 20 min after the end of the mid-week dialysis session [25]. Patients were instructed to take their meals 2 h before dialysis to avoid interference effects of the meal and were not allowed to eat during the course of dialysis.

HD patients were independently evaluated for PEW diagnosis by a different assessor blinded to US measurements; the ISRNM criteria for PEW were used. Briefly, according to ISRNM panel, three out of the four categories, namely serum chemistry; body mass; muscle mass; and dietary intake, must be satisfied for the diagnosis of kidney disease-related PEW [26]. 

Malnutrition inflammation score (MIS) questionnaire was used to assess the degree of malnutrition and inflammation of patients on HD. MIS has four sections including nutritional history, physical examination, BMI and laboratory values; each section receives a score between 0 (normal) to 3 (severely malnourished). Higher scores mean a more severe degree of malnutrition and inflammation [27]. 

### 2.4. Statistical Analysis

Statistical analysis was performed with SPSS (version 23, IBM Corp. Armonk, NY, USA). Data were expressed as means and standard deviations or median and interquartile range (IQR) based on their distribution for continuous variables; and as frequencies (percentage) for categorical variables. ANOVA was used to compare the laboratory differences among three subgroups (MIS score, BMI, and albumin) of HD patients. Differences in muscle and fat thickness between HD patients and controls were adjusted for age, gender, and BMI. The correlation between BIVA parameters and muscle/fat thickness was assessed by Pearson’s correlation coefficient for parametric data and Spearman correlation coefficient for non-parametric data. A multivariable approach was used to assess the association between both the BIVA parameters and QRFM/SFT thicknesses ultrasound, and nutritional indexes (MIS score, BMI, and albumin) of HD patients. Firstly, we tested univariate associations between the MIS score, BMI, and albumin, modeled as continuous variables, and alternatively BIVA or QRFM/SFT parameters by means of linear regression analysis. The variables with *p* < 0.15 at univariate analysis were selected and included in the first multivariate regression model. Next, backward variable selection method with an elimination criterion of *p* < 0.10 was performed to fit the second multivariate linear regression model. 

## 3. Results

Ninety-eight HD patients were screened for enrollment, and 65 patients were enrolled in the study; three patients were excluded for amputation of arts, five patients for prolonged hospitalization within the previous 30 days, seven patients for being bedridden or with immobilization syndrome, nine patients for dialysis vintage less than six months, three patients for presence of cardiac pacemaker, two patients for implantable cardioverter-defibrillator, four patients for denial of consent. 

Thirty-three healthy subjects were also enrolled; HD patients were older than healthy subjects (69 [SD 13.7] vs. 47.2 [SD 7.5] years; *p* < 0.001). No statistically significant differences were found between two groups in the anthropometric parameters (weight: 68 [SD 16.9] vs. 62.4 [SD 12.3] kg, *p* = 0.067; height:165.7 [SD 10.4] vs. 164.9 [SD 6.1] cm, *p* = 0.07; BMI: 24.6 [SD 4.9] vs. 22.9 [SD 3.7] kg/m^2^, *p* = 0.058). 

Demographic, clinical, and nutritional data of HD patients are shown in Table 1. Thirty-eight out of 65 (58.5%) were males, the mean weight loss in the last 6 months was 1.1 Kg (SD 4.1), and serum albumin was 3.7 g/dL (SD 0.4).

HD patients were stratified according to three common nutritional indexes (MIS score, BMI, and albumin) (supplementary Table S1). In summary, albuminemia and TIBC were significantly different between MIS score subgroups (MIS score >6 vs. MIS score <6) (*p* < 0.01). Kt/v was different between the albumin subgroups (albumin >3.8 g/dL vs. albumin <3.8 g/dL) (*p* < 0.001). The differences in MIS score between BMI subgroups (BMI >23 kg/m^2^ vs. BMI <23 kg/m^2^) was not statistically significant (*p* = 0.06). No differences in age and inflammatory status, as assessed by CRP, were found between subgroups. 

**Figure 1:**
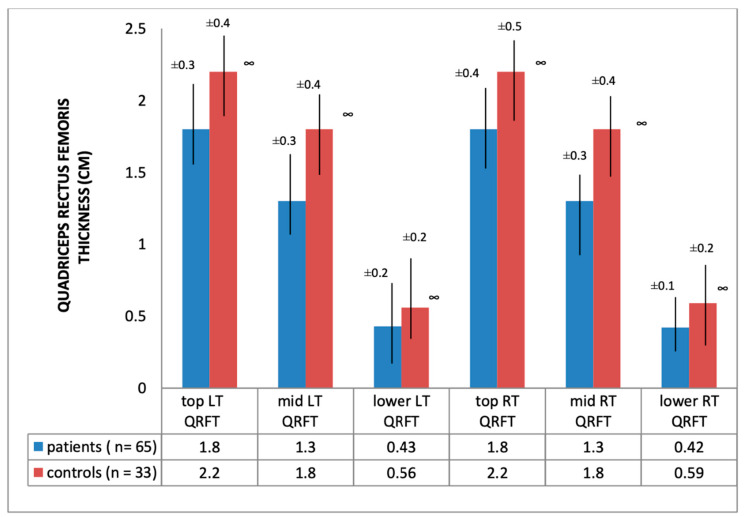
Quadriceps rectus femoris thickness of hemodialysis patients and healthy subjects; ∞ significance = *p* < 0.01; QRFT: Quadriceps rectus femoris thickness; LT: left thigh; RT: right thigh.

**Figure 2:**
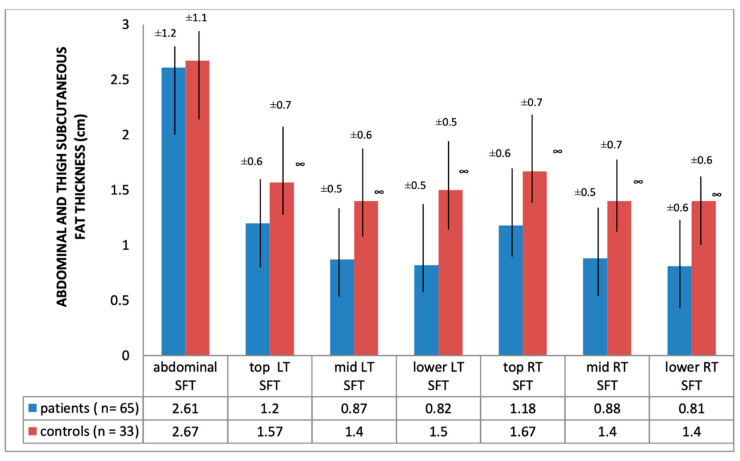
Abdominal/thigh subcutaneous fat thickness of hemodialysis patients and heathy subjects; ∞ significance = *p* < 0.01; SFT: subcutaneous fat tissue; LT: left thigh; RT: right thigh.

Ultrasound thickness of QRFM and SFT were significantly lower at all of the explored sites compared to the control group, except for the abdominal SFT (2.61 [SD 1.2] vs. 2.67 [SD 1.12], *p* = 0.79) (Figure 1 and Figure 2). 

At the multivariate linear regression analysis, after backward selection of variables, with an elimination criterion of *p* < 0.10, abdominal SF and Lower QRFM thickness were significantly associated with BMI, Mid-Thigh SFT thickness was associated with Albumin, whereas Top QRFM thickness was found to predict MIS score β coefficients with 95% confidence intervals are depicted in Table 2. Also, a significant correlation between two nutritional indexes (MIS score and albumin) and some BIVA parameters was found (*p* < 0.05) (Supplementary Table S2).

BIVA parameters were significantly reduced in HD patients compared with healthy subjects (*p* < 0.01), except for Rz and Rz/H. Conversely, extracellular water (ECW) increased (*p* < 0.005) (Table 3). 

The average QRFM thickness of both thigh at top, mid, and lower landmarks were negatively correlated with Rz/H and positively with PA and BCM (*p* < 0.05), whereas the abdominal SFT thickness was significantly and positively correlated with PA, BCM and BMR (Table 4 and Table 5) (supplementary Figures S1 and S2).

## 4. Discussion and Conclusion 

In this study, we showed that QRFM and thigh SFT thicknesses of HD patients, evaluated by the ultrasound technique, were reduced in comparison to healthy subjects and that QRFM thickness was associated with PA, one of the most reliable BIVA parameters also reflecting nutritional status and patients’ prognosis [28].

The reduction in QRFM thickness we observed in HD patients was statistically significant, and also likely to be clinically relevant (more than 20% lower as compared to the control group at each landmark). These results are consistent with the only other study in HD patients [17], in which a significant reduction of rectus femoris and vastus intermedius thickness was found in comparison to both young healthy adults and age-matched hospitalized patients, after adjusting for age, sex and BMI (*p* < 0.01). 

In contrast with this report [17], we did find statistical correlation only between MIS score and Top QRFM thickness. This finding could be explained by some limits of the MIS score: it is partially subjective, it includes albumin, a scarcely sensible variable for nutritional assessment, and a lower non-pathological cut-off value of BMI (>20 kg/m^2^) compared to ISRNM (>23 kg/m^2^) [29]. 

Interesting results emerged regarding fat tissue, thigh SFT thickness was reduced in HD patients in comparison to controls (*p* < 0.001), confirming data obtained in other studies [30,31,32], based on different methods. Conversely, abdominal SFT thickness was not statistically different between the two groups, contradicting the result of other studies in other medical settings in which abdominal SFT, measured by CT, was reduced [33]. However, a partial explanation for this conflicting finding may reside in the fact that the ultrasonographic abdominal fat tissue thickness might be weakly correlated with fat mass in HD patients [34]. 

Besides, analyzing the correlation between thigh and abdominal SFT thickness and nutritional indexes, only the abdominal SFT thickness was associated with BMI. This finding could be explained by the fact that BMI is not able to precisely define body composition, and underestimates both malnutrition and sarcopenic obesity [35]. Sharma et al. showed sarcopenic people, identified by DEXA, were almost all (97%) classified as non-obese by BMI [36].

In the second step of our analysis, we found that HD patients had lower values of BIVA parameters than controls, except for the two parameters that most express intra- and extracellular water volume, namely Rz and Rz/H. Although Rz/H is considered one of discriminant BIVA parameters to identify sarcopenia in elderly individuals of both sexes [37,38], our results are consistent with a recent study in which low accuracy of Rz/H to diagnose malnutrition in HD patients was demonstrated, probably because of the overhydration status of patients [27]. 

A further analysis of our study showed a statistically significant correlation between QRFM thickness and BIVA parameters, namely PA and BCM, the BIVA surrogate parameters of muscle mass and that allow the identification of sarcopenia in HD patients [39,40]. 

Indeed, phase angle is related to cell integrity and considered a prognostic indicator of nutritional risk in HD patients [41,42]. Low PA indicates muscle loss, although cut-off points to identify malnourished individuals are missing [43]. Similarly, body cell mass, derived from the calculation of total body potassium [44], is considered a valid index of skeletal muscle mass, as it represents the metabolic active part of cell mass. 

On the other hand, no correlations were found between thigh SFT and BIVA parameters. These findings could be explained by the fact that BIVA is not very accurate to evaluate the fat mass [45].

The strength of this study is that it reported, for the first time, the association between QRFM and SFT thickness evaluated by US and BIVA parameters in HD patient. The integration of US with BIVA, in the clinical practice, can have potential benefits since their synergy, providing an early and accurate identification of malnourished HD patients at bedside, might reduce the rate of morbidity and mortality of HD patients [46]. Although ultrasound is an easy and quick technique to visualize the fat and muscle mass [20,47], a dedicated education and accurate practical training are required in order to reduce diagnostic errors due to the operator dependent imaging modality and the lack of validated reference value for QRFM and SFT thickness.

There are, however, some limitations in our study that also should be mentioned. First, the small sample size of our population does not allow us to generalize our results. Further multicenter studies on larger samples of HD patients should be considered for future research. Another limitation is represented by the fact that ultrasound measurement of the subcutaneous fat was performed at four landmarks, less than those recently proposed [48,49] to fully evaluate the fat mass; however, this new evidence was not available yet when the present study was done. In addition, we did not calculate the body cell mass index, a more reliable index for the evaluation of body composition quality, being the sample too small for a correct analysis. Finally, we did not take into account the level of physical activity, a relevant variable of muscle mass [50,51,52,53,54,55]. However, available data strongly demonstrate that HD patients are prevalently sedentary [56,57,58,59]

In conclusion, our study demonstrates that ultrasound is a valid tool to identify HD patients with significant reduction of quadriceps rectus femoris muscle and subcutaneous fat tissue thicknesses, and that muscle ultrasound measurements have good agreement with BIVA parameters. Ultrasound should be considered a practical, easy, and cheap tool that provides a fast analysis of muscle and fat mass. It could be integrated with other currently available simple techniques, such as BIVA, early identification of PEW and for nutritional status monitoring of HD patients. 

## Figures and Tables

**Table 1:** Demographic, and clinical data of hemodialysis patients.

Socio-Demographic Variables		Clinical Variables	
Age, years *	69 (13.7)	Systolic Blood Pressure, mm hg *	138.3 (22.8)
Sex, Males, *n* (%)	38 (58.5)	Diastolic Blood Pressure, mm hg *	73.4 (11.8)
Weight loss, last 6 months **	−1.0 (−2.5–0.45)	Heart Rate, bpm *	68.4 (9.4)
		Height, cm *	165.7 (10.4)
Diabetes mellitus, %	29.2	Weight, kg *	68 (16.9)
Caucasian race, %	98.5	Body Mass Index, kg/m^2^ *	24.6 (4.9)
Diabetes mellitus, %	29.2		
Previous Stroke, %	20	Blood Test Values	
COPD, %	18.5	Serum phosphorus, mg/dL *	6.1 (2.5)
Cardiovascular diseases, %	36.9	Serum calcium, mg/dL *	9.3 (0.8)
PAD, %	30.8	PTH, pg/mL **	217 (116.5–377)
Previous renal transplantation, %	9.2	Albumin, g/dL *	3.7 (0.4)
History of cancer, %	30.8	Ferritin, microg/L **	285 (80.5–491.5)
		Transferrin, mg/dL *	188.4 (43.5)
Kidney disease:		Serum iron, microg/dL *	57.8 (29.3)
Glomerulonephritis, %	20	Total Iron Binding Capacity, mg/dL *	235.5 (54.3)
Nephroangiosclerosis, %	16.9	Total Cholesterol, mg/dL *	161.3 (47.4)
ADPKD, %	4.6	HDL Cholesterol, mg/dL *	39.9 (11.2)
Others, %	26.2	Triglycerides, mg/dL **	140 (103–197)
		C-reactive Protein, mg/dL **	0.46 (0.19–0.85)
		KT/V	1.4 (0.3)

* Data are expressed as means (standard deviations); ** Data are expressed as median and range Interquartile; ADPKD: Autosomal dominant polycystic kidney disease; COPD: chronic obstructive pulmonary disease; HDL: high-density lipoprotein; PAD: peripheral artery disease; PTH: parathormone.

**Table 2:** Correlation between SFT / QRTM thickness and nutritional indexes (BMI, albumin, MIS score) in hemodialysis patients.

Dependent Variable: BMI							
Model	UC		SC	*t*	Sig.	95 % CI	
	B	St. Error	Beta			LB	UB
(Constant)	13.369	2.448		5.461	0.000	8.470	18.267
Abdominal SFT thickness	1.341	0.507	0.323	2.646	0.010	0.327	2.356
Lower QRFM thickness	10.173	3.067	0.350	3.317	0.002	4.036	16.310
**Dependent Variable: MIS Score**							
**Model**	**UC**		**SC**	***t***	**Sig.**	**95 % CI**	
	**B**	**St. Error**	**Beta**			**LB**	**UB**
(Constant)	18.141	2.713		6.688	0.000	12.713	23.569
Mid Thigh SFT thickness	3.381	1.841	0.343	1.837	0.071	−0.303	7.064
Lower Thigh SFT thickness	−3.584	2.004	−0.339	−1.788	0.079	−7.594	0.427
Top QRFM thickness	−4.432	1.320	−0.377	−3.358	0.001	−7.073	−1.791
Lower QRFM thickness	−6.007	3.221	−0.208	−1.865	0.067	−12.453	0.439
**Dependent Variable: Albumin**							
**Model**	**UC**		**SC**	***t***	**Sig.**	**95 % CI**	
	**B**	**St. Error**	**Beta**			**LB**	**UB**
(Constant)	3.480	0.224		15.506	0.000	3.031	3.929
Mid Thigh SFT thickness	−0.489	0.152	−0.622	−3.211	0.002	−0.794	−0.184
Top QRFM thickness	0.195	0.109	0.208	1.789	0.079	−0.023	0.414

BMI: Body Mass Index; CI: Confidence Interval; LB: Lower Bound; LT: Left Thigh; MIS: Malnutrition Inflammation Score; PA: Phase Angles; QRFM: Quadriceps Rectus Femoris Muscle; RT: Right Thigh; SC: Standardized Coefficients; SFT: Subcutaneous Fat Tissue; UB: Upper Bound; UC: Unstandardized Coefficients.

**Table 3:** Bioelectrical impedance analysis parameters of hemodialysis patients and heathy subjects.

	Patients (*n* = 65)	Controls (*n* = 33)	*p*
Rz	545.7 (82.9)	569.5 (69.6)	0.63
Xc	42.8 (11.3)	60.3 (9.3)	0.001
Rz/H	3.3 (0.58)	3.5 (0.5)	0.46
Xc/H	0.26 (0.07)	0.4 (0.07)	0.003
BCM	19.8 (8.3)	24.4 (5.9)	<0.001
BMR KJOULE	5362.9 (1262.9)	6203.5 (522.7)	0.007
BMR KCAL	1262.5 (340.1)	1482.7 (124.9)	<0.01
ECW	29.2 (15.9)	15.3 (3.3)	0.005
PHASE ANGLE	4.5 (1.2)	6.1 (0.8)	<0.001

Data are expressed as means (standard deviations); BCM: Body cell mass; BMR: basic metabolic rate; ECW: extra-cellular mass; H: height; Rz: resistance; Xc: reactance.

**Table 4:** Correlation between quadriceps rectus femoris thickness and BIVA parameters in hemodialysis patients.

	RZ	RZ/H	XC	XC/H	BCM	BMR KJOULE	PHASE ANGLE
**TOP QRFM THICKNESS (P−VALUE)**	−0.236(0.058)	−0.319 (0.010) *	0.125 (0.322)	0.055 (0.661)	0.12 (0.011) *	0.355 (0.004) *	0.315 (0.011) *
**MID QRFM THICKNESS (P−VALUE)**	−0.257 (0.039) *	−0.264(0.034) *	0.11 (0.383)	0.093 (0.461)	0.258 (0.038) *	0.294 (0.018) *	0.232 (0.043) *
**LOWER QRFM THICKNESS (P−VALUE)**	−0.239 (0.057)	−0.273 (0.029) *	−0.280 (0.025)	0.212 (0.092)	0.365 (0.003) *	0.128 (0.312)	0.423 (<0.001) *

* significance = *p* < 0.05; BCM: Body cell mass; BMR: basic metabolic rate; H: height; QRFM: quadriceps rectus femoris; Rz: resistance; Xc: reactance.

**Table 5:** Correlation between abdominal/thigh subcutaneous fat thickness and BIVA parameters in hemodialysis patients.

	Rz	Rz/H	Xc	Xc/H	BCM	BMR KJOULE	PHASE ANGLE
Abdominal SFT thickness (*p*−value)	−0.148 (0.239)	−0.204 (0.102)	0.218 (0.081)	0.161 (0.200)	0.294 (0.018) *	0.303 (0.014) *	0.299 (0.016) *
Top Thigh SFT thickness (*p*−value)	−0.27 (0.83)	0.036 (0.778)	−0.001 (0.992)	0.033 (0.793)	0.013 (0.919)	−0.041 (0.746)	−0.034 (0.785)
Mid Thigh SFT thickness (*p*−value)	0.014 (0.91)	0.161 (0.2)	−0.055 (0.661)	0.043 (0.732)	−0.211 (0.092)	−0.119 (0.346)	−0.087 (0.493)
Lower Thigh SFT thickness (*p*−value)	−0.088 (0.487)	−0.018 (0.886)	0.068 (0.588)	0.212 (0.089)	0.066 (0.601)	0.160 (0.204)	0.225 (0.071)

* significance = *p* < 0.05; BCM: Body cell mass; BMR: basic metabolic rate; H: height; Rz: resistance; SFT: subcutaneous fat tissue; Xc: reactance.

## Supplementary Materials

The following are available online at https://www.mdpi.com/2072-6643/12/5/1388/s1, Figure S1: Correlation between QRFM thickness and BIVA parameters, Correlation between Abdominal SFT thickness and BIVA parameters, Table S1: Clinical metabolic and nutritional data of hemodialysis patients stratified by BMI, albumin and MIS score, Table S2: Correlation between BIVA parameters and nutritional indexes (MIS score, albumin and BMI) in HD patients.

## Abbreviations

BCM	Body Cell Mass
BMI	Body Mass Index
BIVA	Bioelectrical Impedance Vector Analysis
CRP	C-reactive Protein
CT	Computed Tomography
DEXA	Dual-Energy X-ray Absorptiometry
ECM	Extra-Cellular Mass
H	Height
HD	Hemodialysis
ISRNM	International Society of Renal Nutrition and Metabolism
MIS	Malnutrition Inflammation Score
PEW	Protein Energy Wasting
QRFM	Quadriceps Rectus Femoris Muscle
Rz	Resistance
SFT	Subcutaneous Fat Tissue
US	Ultrasound
Xc	Reactance
