# Supplementary material for: Ultrasonography of Quadriceps Femoris Muscle and Subcutaneous Fat Tissue and Body Composition by BIVA in Chronic Dialysis Patients
Source: Nutrients. 2020 May 12;12(5):1388. doi: 10.3390/nu12051388 (PMC7285004; doi:10.3390/nu12051388)
Supplement: Supplementary file 1 [file nutrients-12-01388-s001.pdf]

# SUPPLEMENTARY FIGURE AND TABLE

**Table S1**: Clinical. metabolic and nutritional data of hemodialysis patients stratified by BMI, albumin and MIS score.

| | BMI | | | Albumin | | | MIS score | | |
|---|---|---|---|---|---|---|---|---|---|
| | < 23 Kg/m$^2$ | > 23 Kg/m$^2$ | | < 3.8 g/dl | > 3.8 g/dl | | < 6 score | > 6 score | |
| | (n= 25) | (n=40) | p | (n=34) | (n=31) | p | (n=25) | (n=40) | p |
| **Age, years *** | 68 (17) | 69 (11.8) | 0.42 | 70.8 (13.4) | 67 (14) | 0.27 | 64.9 (14) | 71.5 (13) | 0.62 |
| **Sex, Males, n (%)** | 45 | 40 | 0.71 | 52.9 | 29 | 0.51 | 24 | 52.5 | 0.30 |
| **Caucasian race, %** | 100 | 97.5 | 0.47 | 100 | 96.7 | 0.29 | 100 | 97.5 | 0.43 |
| **Diabetes mellitus, %** | 30 | 35 | 0.11 | 26.4 | 32 | 0.61 | 24 | 32.5 | 0.46 |
| **Previous Stroke, %** | 20 | 25 | 0.59 | 14.7 | 25.8 | 0.26 | 16 | 22.5 | 0.52 |
| **COPD, %** | 25 | 22.5 | 0.49 | 11.7 | 25.8 | 0.15 | 20 | 17.5 | 0.80 |
| **Cardiovascular diseases, %** | 30 | 35 | 0.71 | 41.2 | 32 | 0.46 | 24 | 45 | 0.88 |
| **PAD, %** | 30 | 32.5 | 0.84 | 38.2 | 22.5 | 0.17 | 24 | 35 | 0.35 |
| **Previous renal transplantation, %** | 10 | 50 | 0.19 | 8.8 | 12.9 | 0.33 | 9 | 10 | 0.79 |
| **History of cancer, %** | 40 | 35 | 0.70 | 29.4 | 32 | 0.80 | 32 | 30 | 0.87 |
| **MIS < 6 score, %** | 25 | 17 | 0.06 | 14.7 | 64.5 | 0.37 | 100 | 0 | <0.001 |
| **Systolic Blood Pressure, mmHg*** | 139.8 (21) | 136.4 (23.7) | 0.60 | 134.5 (24.9) | 142.3 (20) | 0.17 | 140 (21.6) | 137.2 (23.1) | 0.63 |
| **Diastolic Blood Pressure, mmHg*** | 75.8 (14.9) | 72 (10.2) | 0.32 | 70.5 (9.9) | 76.6 (13) | 0.40 | 77.5 (10.5) | 70.9 (12) | 0.20 |
| **Heart Rate, bpm*** | 68.9 (7.9) | 68.8 (10.4) | 0.24 | 68.4 (8.4) | 68.4 (10.5) | 0.98 | 70.5 (8) | 67 (10) | 0.13 |
| **KT/V*** | 1.4 (0.27) | 1.3 (0.6) | 0.74 | 1.5 (0.26) | 1.3 (0.24) | <0.001 | 1.3 (0.24) | 1.4 (0.27) | 0.30 |
| **Serum phosphorus, mg/dl*** | 5.4 (2.1) | 6.2 (2.4) | 0.61 | 5.8 (2.9) | 6.4 (1.9) | 0.31 | 6.8 (2.3) | 5.7 (2.5) | 0.57 |
| **Serum calcium, mg/dl*** | 9.3 (0.9) | 9.3 (0.73) | 0.23 | 9.3 (0.9) | 9.3 (0.7) | 0.91 | 9.3 (0.74) | 9.3 (0.86) | 0.87 |
| **PTH, pg/ml**** | 194 (68-347) | 205.5 (120.2-400.2) | 0.74 | 180.5 (91-300.5) | 308 (146-465) | 0.11 | 299 (159.5-415.5) | 172.5 (85-330.5) | 0.28 |
| **Albumin, g/dL*** | 3.6 (0.4) | 3.8 (0.4) | 0.10 | 3.4 (0.7) | 4 (0.20) | <0.001 | 4 (0.7) | 3.5 (0.34) | <0.001 |
| **Ferritin, microg/L**** | 250 (56-510.5) | 295 (80.5-501.2) | 0.74 | 348 (115.2-575) | 218 (68-425) | 0.25 | 189 (50-379.5) | 373.5 (123.5-557) | 0.53 |

| | | | | | | | | | |
|---|---|---|---|---|---|---|---|---|---|
| **Serum iron, microg/dL*** | 48.9 (25.6) | 62.8 (31.6) | 0.76 | 54 (30) | 61.8 (28.5) | 0.30 | 56.8 (23.9) | 58.3 (32.6) | 0.82 |
| **Total Iron Binding Capacity, mg/dL*** | 217.8 (49.1) | 243.9 (58.5) | 0.14 | 226.7 (55.1) | 247.2 (52.6) | 0.17 | 260.65 (49.3) | 219.8 (51.9) | <0.001 |
| **Total Cholesterol, mg/dL*** | 156.6 (48.9) | 162.3 (104.8) | 0.13 | 159.3 (48) | 163.5 (47.5) | 0.72 | 165.9 (47.3) | 158.5 (47.8) | 0.54 |
| **HDL Cholesterol, mg/dL*** | 39.1 (10) | 39 (11.5) | 0.29 | 40.7 (13.6) | 39 (7.9) | 0.54 | 40.8 (10.9) | 39.4 (11.5) | 0.60 |
| **Triglycerides, mg/dL**** | 121.5 (88-184.5) | 160.5 (113.2-234.7) | 0.32 | 140 (90.5-195) | 143 (114-209) | 0.71 | 143 (109.5-198) | 139 (94.5-199) | 0.94 |
| **C-reactive Protein, mg/dL**** | 0.53 (0.26-1.19) | 0.46 (0.19-0.93) | 0.52 | 0.47 (0.21-1.37) | 0.45 (0.17-0.79) | 0.06 | 0.44 (0.18-0.76) | 0.51 (0.19-1.31) | 0.12 |

*Data are expressed as means (standard deviations); ** Data are expressed as median and range Interquartile; ADPKD: Autosomal dominant polycystic kidney disease; COPD: chronic obstructive pulmonary disease; PAD: peripheral artery disease; PTH: parathormone.

**Fig. S1**. Correlation between QRFM thickness and BIVA parameters

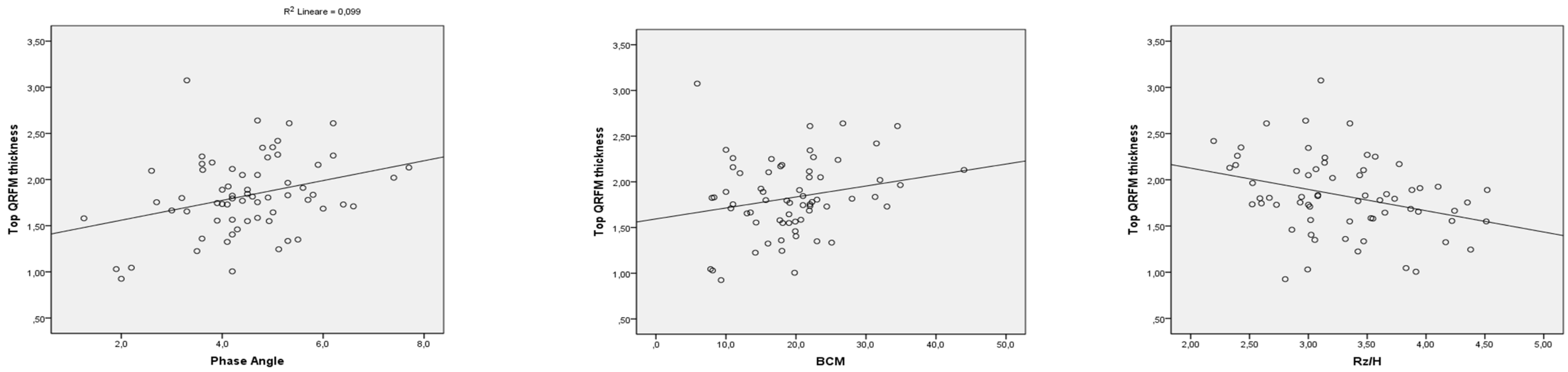

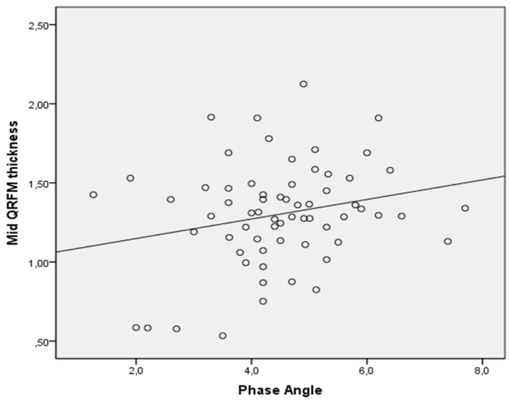
Mid QRFM thickness
,50
1,00
1,50
2,00
2,50
2,0
4,0
6,0
8,0
Phase Angle

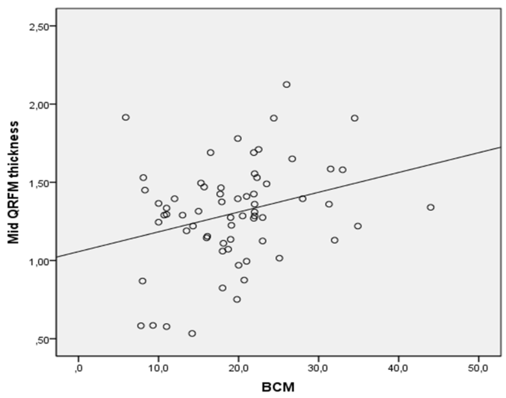
Mid QRFM thickness
,50
1,00
1,50
2,00
2,50
,0
10,0
20,0
30,0
40,0
50,0
BCM

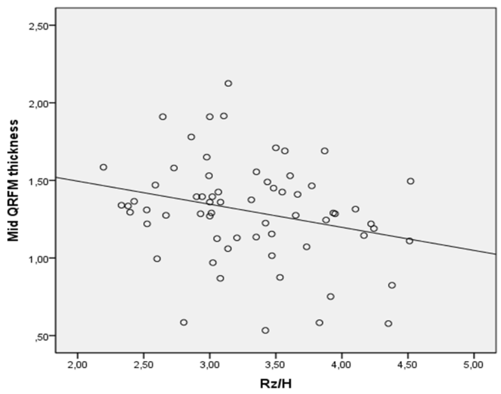
Mid QRFM thickness
,50
1,00
1,50
2,00
2,50
2,00
2,50
3,00
3,50
4,00
4,50
5,00
Rz/H

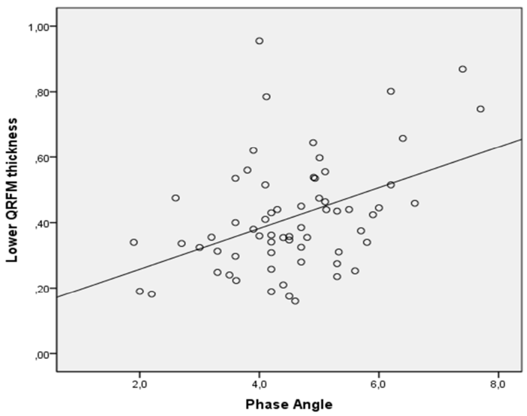
Lower QRFM thickness
,00
,20
,40
,60
,80
1,00
2,0
4,0
6,0
8,0
Phase Angle

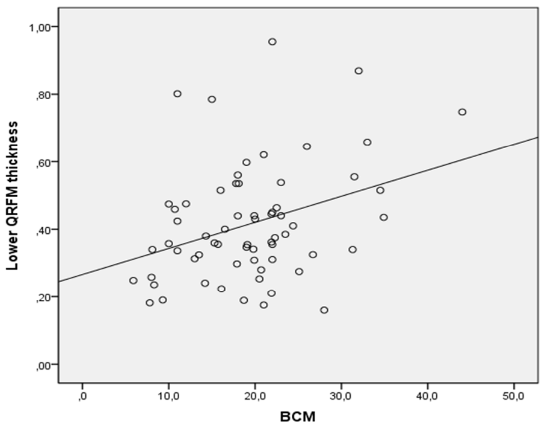
Lower QRFM thickness
,00
,20
,40
,60
,80
1,00
,0
10,0
20,0
30,0
40,0
50,0
BCM

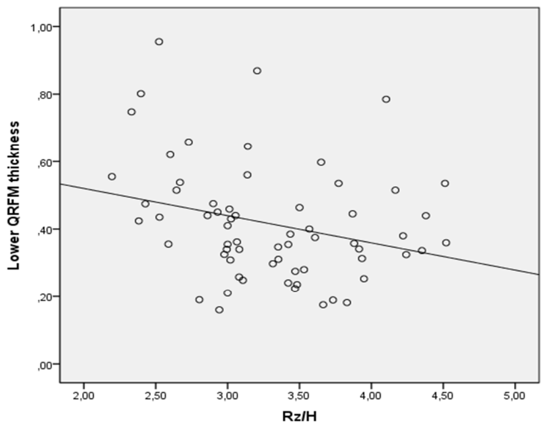
Lower QRFM thickness
,00
,20
,40
,60
,80
1,00
2,00
2,50
3,00
3,50
4,00
4,50
5,00
Rz/H

**Fig. S2**. Correlation between Abdominal SFT thickness and BIVA parameters

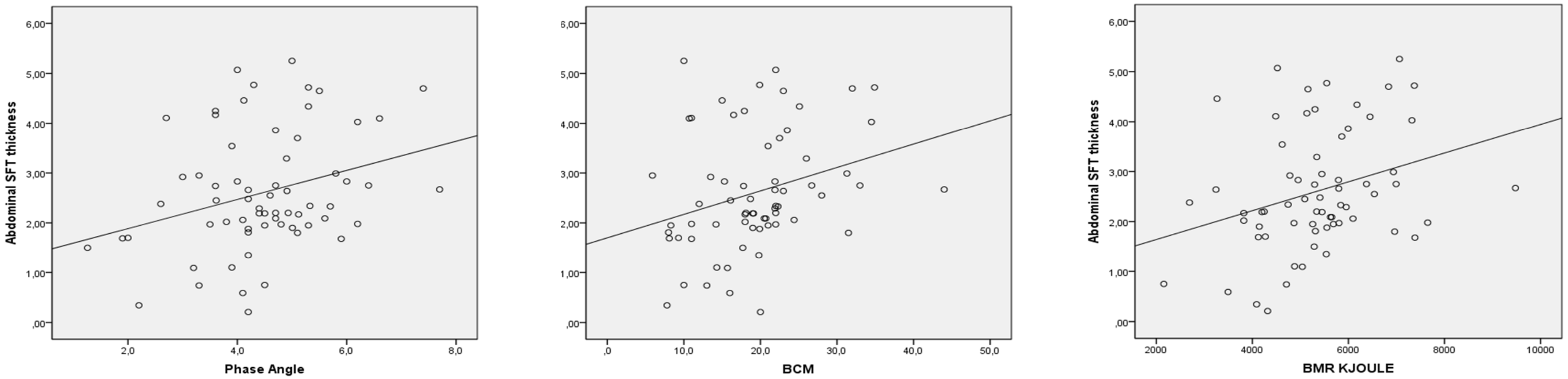


**Table S2**. Correlation between BIVA parameters and nutritional indexes (MIS score, albumin and BMI) in HD patients.

| | Dependent Variable: MIS Score | | | | | | | Dependent Variable: Albumin | | | | | | | Dependent Variable: BMI | | | | | | |
|---|---|---|---|---|---|---|---|---|---|---|---|---|---|---|---|---|---|---|---|---|---|
| | UC | | SC | | | 95 % CI | | UC | | SC | | | 95 % CI | | UC | | SC | | | 95 % CI | |
| Model | B | St. Error | Beta | t | Sig. | LB | UB | B | St. Error | Beta | t | Sig. | LB | UB | B | St. Error | Beta | t | Sig. | LB | UB |
| (Costant) | -3,156 | 6,653 | | -,474 | ,637 | -16,470 | 10,158 | 3,561 | ,416 | | 8,555 | ,000 | 2,728 | 4,393 | 36,301 | 5,061 | | 7,172 | ,000 | 26,177 | 46,424 |
| Rz/H | 6,398 | 1,807 | ,753 | 3,540 | ,001 | 2,782 | 10,014 | -,162 | ,084 | -,237 | -1,933 | ,058 | -,329 | ,006 | -3,773 | 1,018 | -,441 | -3,706 | ,000 | -5,809 | -1,736 |

| | | | | | | | | | | | | | | | | | | | | | |
|---|---|---|---|---|---|---|---|---|---|---|---|---|---|---|---|---|---|---|---|---|---|
| Xc/H | - 58,813 | 19,734 | -,831 | -2,980 | ,004 | -98,300 | -19,326 | | | | | | | | | | | | | | |
| BCM | | | | | | | | | | | | | | | ,265 | ,091 | ,401 | 2,918 | ,005 | ,083 | ,446 |
| BMR KJOULE | | | | | | | | ,000 | ,000 | ,606 | 2,491 | ,016 | ,000 | ,000 | | | | | | | |
| BMR KCAL | | | | | | | | -,001 | ,000 | -,520 | -1,977 | ,053 | -,001 | ,000 | | | | | | | |
| PA | | | | | | | | ,097 | ,048 | ,303 | 2,010 | ,049 | ,000 | ,194 | | | | | | | |

BMI: Body Mass Index; BCM: Body cell mass; BMR: basic metabolic rate; ; CI: Confidence Interval; H: height; LB: Lower Bound; MIS: Malnutrition Inflammation Score; PA: Phase Angle; Rz: resistance; SC: Standardized Coefficients; UB: Upper Bound; UC: Unstandardized Coefficients; Xc: reactance.
